# Scalable Infrastructure Supporting Reproducible Nationwide Healthcare Data Analysis toward FAIR Stewardship

**DOI:** 10.1038/s41597-023-02580-7

**Published:** 2023-10-04

**Authors:** Ji-Woo Kim, Chungsoo Kim, Kyoung-Hoon Kim, Yujin Lee, Dong Han Yu, Jeongwon Yun, Hyeran Baek, Rae Woong Park, Seng Chan You

**Affiliations:** 1 Big Data Department, Health Insurance Review and Assessment Service, Wonju, Republic of Korea; 2 Department of Biomedical Sciences, Ajou University Graduate School of Medicine, Suwon, Republic of Korea; 3 Review and Assessment Research Department, Health Insurance Review and Assessment Service, Wonju, Republic of Korea; 4 Department of Biomedical Informatics, Ajou University School of Medicine, Suwon, Republic of Korea; 5 Department of Biomedical Systems Informatics, Yonsei University College of Medicine, Seoul, Republic of Korea; 6 Institution for Innovation in Digital Healthcare, Yonsei University, Seoul, Republic of Korea

**Keywords:** Epidemiology, Outcomes research, Databases, Epidemiology

## Abstract

Transparent and FAIR disclosure of meta-information about healthcare data and infrastructure is essential but has not been well publicized. In this paper, we provide a transparent disclosure of the process of standardizing a common data model and developing a national data infrastructure using national claims data. We established an Observational Medical Outcome Partnership (OMOP) common data model database for national claims data of the Health Insurance Review and Assessment Service of South Korea. To introduce a data openness policy, we built a distributed data analysis environment and released metadata based on the FAIR principle. A total of 10,098,730,241 claims and 56,579,726 patients’ data were converted as OMOP common data model. We also built an analytics environment for distributed research and made the metadata publicly available. Disclosure of this infrastructure to researchers will help to eliminate information inequality and contribute to the generation of high-quality medical evidence.

## Introduction

Numerous studies using routinely collected large healthcare data have provided invaluable evidence representing routine clinical practice^1,2^. Administrative data representing the nationwide population have been used for secondary analysis in healthcare research for various purposes, including consecutive monitoring of disease and medical expenditure, comparative effectiveness of medical interventions, and even machine learning^3–6^. The Korean National Health Insurance system is a single public insurance system for all citizens, and all medical institutions are applied as mandatory designation systems. The Health Insurance Review and Assessment Service (HIRA) establishes health insurance reimbursement criteria and reviews all medical claims for reimbursement. Therefore, the HIRA has accumulated a vast amount of claims data at the national level, and it can be used as a secondary data source for high-quality real-world evidence^7^. For example, statistics from the HIRA database are used in OECD statistics as representative statistics for Korea.

Administrative data, despite being a commonly used source for research, has drawn significant criticism predominantly due to concerns over the validity of its coded information. For instance, coding practices like “upcoding” can lead to inaccuracies; this is where providers code for a more severe illness than the patient actually has to receive higher reimbursement^8,9^. While the debate on coded information’s validity continues, less attention is being directed towards the stewardship of this extensive healthcare data. Chief among these are issues including: 1. Non-scalability and non-interoperability; 2. Ignored reproducibility; and 3. Protection of privacy of the national population. Such areas might pose even more profound implications for the utility and reliability of large healthcare datasets.

A distributed research system based on a common data model has emerged as a promising alternative to address the concerns surrounding the use of large healthcare datasets^10^. The Observational Medical Outcome Partnership Common Data Model (OMOP-CDM) is a standardized data model maintained by Observational Health Data Sciences and Informatics (OHDSI), which is a global, multi-stakeholder, interdisciplinary community. The OMOP-CDM was designed to enable the systematic analysis of large observational datasets from multiple data sources by providing a common structure and vocabulary for observational data. In response to the urgent requirement for coronavirus disease-2019 (COVID-19) research, the HIRA was the first institution in the world to standardize the data of patients with COVID-19 into OMOP-CDM, providing access to international researchers without compromising patient privacy^11^. This approach inspired other database owners, enabling researchers to conduct multiple high impact studies using the multi-national database in a timely manner^12^. However, thus far, the HIRA database has been standardized to OMOP-CDM for individual studies, and standardized data have not been maintained^13^.

We aimed to standardize HIRA data into OMOP-CDM, build infrastructure providing scalable accessibility and a flexible data analysis environment with privacy-by-design protection, and verify whether the infrastructure guarantees the reproducibility of research. The aim of this study was to enhance the FAIRness of the national healthcare database, which refers to its ability to be easily Findable, Accessible, Interoperable, and Reusable (FAIR)^14^. Specifically, in this study, the process of converting national claims data into research data to establish research infrastructure, mapping local code to standard vocabulary system, verifying data through type 2 diabetes mellitus (T2DM) cases and replicating previously published COVID-19 prediction study. In addition, external disclosure of the infrastructure by the FAIR principle was reviewed.

## Results

### Basic statistics of HIRA CDM

We extracted, transformed, and loaded (ETL) the HIRA database into the OMOP-CDM version 5.3.1. All tables specified by the OMOP-CDM conversion specifications were created. The number of converted claims specification and number of patients included were 10,098,730,241 and 56,579,726, respectively (Table 1). Among the converted data, the number of males and females was 28,439,311 (50.3%) and 28,140,325 (49.7%), respectively. All records of the source database were converted into CDM format without errors in classification by year, type of visit, and type of claiming medical institution (Table S1 in the Supplements). Among the CDM tables, the death table contained information of 3,804,948 people who had died over 11 years, accounting for 6.7% of the total population (Table 1). The condition, drug, and procedure tables, which are the main clinical information of the OMOP-CDM, included more than 99.0% of patients, and devices and measurements included more than 90.0% of patients (Table 1).

**Table 1:** Number of records, number of persons, and their ratio in HIRA CDM database.

OMOP-CDM tables	Records (n)	Person (n)	Person/total person (%)
CARE SITE	213 758	0	0.0
CONDITION ERA	12 600 281 758	56 536 873	99.9
CONDITION OCCURRENCE	26 798 208 704	56 536 873	99.9
COST	76 131 071 211	0	0.0
DEATH	3 804 948	3 804 948	6.7
DEVICE EXPOSURE	892 251 206	51 913 063	91.8
DRUG ERA	15 052 413 048	56 363 138	99.6
DRUG EXPOSURE	28 732 916 071	56 389 812	99.7
MEASUREMENT	10 190 277 150	53 603 975	94.7
OBSERVATION	499 711 003	40 949 894	72.4
OBSERVATION PERIOD	56 579 726	56 579 726	100.0
PAYER PLAN PERIOD	56 579 726	56 579 726	100.0
PERSON	56 579 726	56 579 726	100.0
PROCEDURE OCCURRENCE	27 683 994 844	56 547 684	99.9
VISIT OCCURRENCE	10 098 730 241	56 579 728	100.0

HIRA: Health Insurance Review and Assessment Service; OMOP: Observational Medical Outcome Partnership; CDM: common data model; n: number.

The results of vocabulary mapping from the Electronic Data Interchange (EDI) codes of Korea to the OMOP standardized vocabulary are shown in Table 2. Table 2 lists the number of EDI codes according to the OMOP domain, ratio of codes mapped to standard terminologies, and number of mapped records per source record. Regarding the ratio of mapped codes to source codes, condition (99.1%), drug (100.0%), observation (99.97%), and procedure (84.5%) were high, however, device (10.8%) and measurement (31.0%) were relatively low. However, the ratio of mapped records (mapped records per source records) was over 85.0% in all domains including device (87.6%) and measurement (91.5%).

**Table 2:** Status of vocabulary mapping in the converted HIRA CDM.

Contents	OMOP-CDM tables					
	CONDITION OCCURRENCE	PROCEDURE OCCURRENCE	DRUG EXPOSURE	DEVICE EXPOSURE	MEASUREMENT	OBSERVATION
Source code, n	19 084	322 136	63 095	20 082	22 765	3 481
Mapped code, n	18 910	272 163	63 095	2 159	7 049	3 480
Mapped code ratio, %	99.1	84.5	100.0	10.8	31.0	99.97
Source records, n	26 798 208 704	27 683 994 844	28 732 916 071	892 251 206	10 190 277 150	499 711 003
Mapped records, n	26 599 002 701	26 111 671 178	28 692 327 376	781 209 308	9 326 266 819	499 596 519
Mapped records ratio, %	99.3	94.3	99.9	87.6	91.5	99.98

HIRA: Health Insurance Review and Assessment Service; CDM: common data model; OMOP: Observational Medical Outcome Partnership;

### Data quality and reliability

We compared the amount of original (source) and converted data for the condition/drug/procedure/device codes. The number of records from the source and converted data and their differences from the top 10 codes in each domain are presented in the Tables S2–S6 in the Supplements. The differences were due to (1) the multiple mapping of the source code, (2) the assignment to a different domain table from the source table, and (3) the absence of mapping to OMOP standardized vocabulary.

**Fig. 1:**
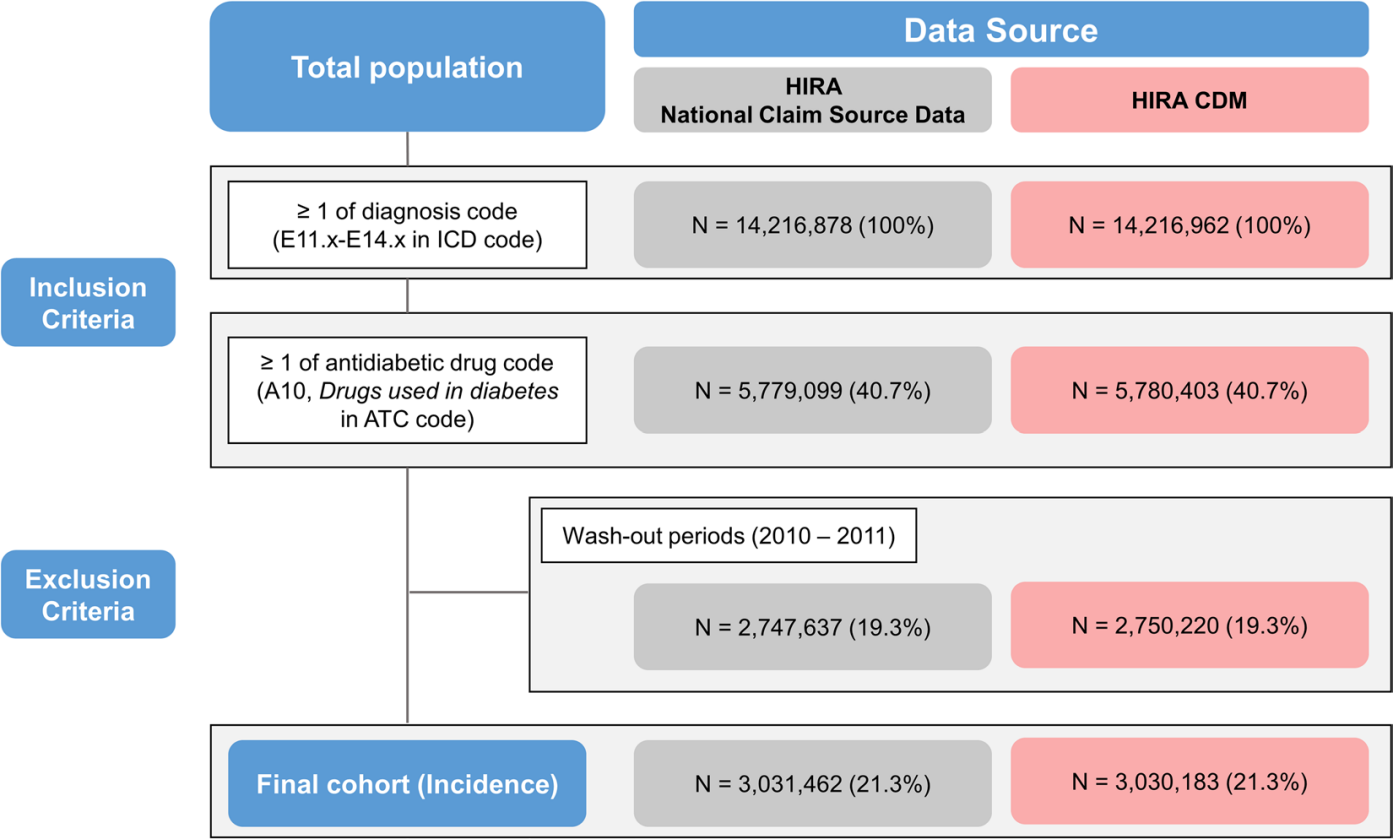
Flow chart of type 2 diabetes mellitus phenotype and comparison of incidences from the source and converted CDM databases. CDM: common data model.

The number of patients with T2DM was extracted according to the same definition from the source and converted data, and the numbers of patients were 3,031,462 (21.3%) and 3,030,183 (21.3%), respectively (Fig. 1). The incidence of T2DM per 100,000 patients ranged from 550.1 to 650.9 and 549.9 to 649.7 in the source and converted HIRA CDM database, respectively (Table 3). In 2012, the difference in the number of patients with T2DM between the source and converted data was 590, and the difference in the incidence rate was the largest at 1.2 per 100,000 patients. The difference in the number of patients was 14, and the difference in the incidence rate was 0.0 in 2020. In addition, there were no differences in T2DM incidence by year-gender and year-age groups (Tables S7, S8 in the Supplements).

**Table 3:** Incidence and difference of type 2 diabetes mellitus phenotype by year between source and converted HIRA CDM data.

Year	Population size at the middle of each year	HIRA Source data		HIRA CDM		Differences	
		N	Incidence*	N	Incidence*	N	Incidence*
		(A)	(B)	(C)	(D)	(A)-(C)	(B)-(D)
2012	50 199 853	326 732	650.9	326 142	649.7	590	1.2
2013	50 428 893	304 986	590.4	304 594	589.7	392	0.7
2014	50 746 659	291 837	550.1	291 750	549.9	87	0.2
2015	51 014 947	304 215	558.7	304 784	559.8	−569	−1.1
2016	51 217 803	337 933	607.7	337 561	607.0	372	0.7
2017	51 361 911	350 511	617.0	350 327	616.7	184	0.3
2018	51 585 058	356 570	615.1	356 420	614.8	150	0.3
2019	51 764 822	374 174	631.1	374 115	631.0	59	0.1
2020	51 836 239	384 504	636.6	384 490	636.6	14	0.0

*Age/sex-standardized incidence rate per 100 000 standard fixed population. Fixed population was referenced from KOSIS, Statistics Korea. HIRA: Health Insurance Review and Assessment service; CDM: common data model, N: number.

In the HIRA CDM database, by 2020, 32,633 outpatients were diagnosed with COVID-19. We could validate a previously published COVID-19 prediction model (COVER model) which developed based on the OMOP-CDM^15^. The performance of the COVER models to predict hospitalization for pneumonia, admission to the ICU or death from pneumonia, and all-cause death were 0.816, 0.891, and 0.892, respectively (Table S9 in the Supplements). We also tried to validate the models using newly updated sampled database. The HIRA 20% sample database until April 2022, 1,530,350 outpatients were diagnosed with COVID-19, and the performance of the model was 0.748 (hospitalization for pneumonia), 0.879 (admission to the ICU with pneumonia or death due to pneumonia), and 0.891 (all-cause mortality). Through version control of the database, we confirmed that predictive models developed earlier could be easily applied to databases of different versions with different periods.

### Data analytic environment and open policy

**Fig. 2:**
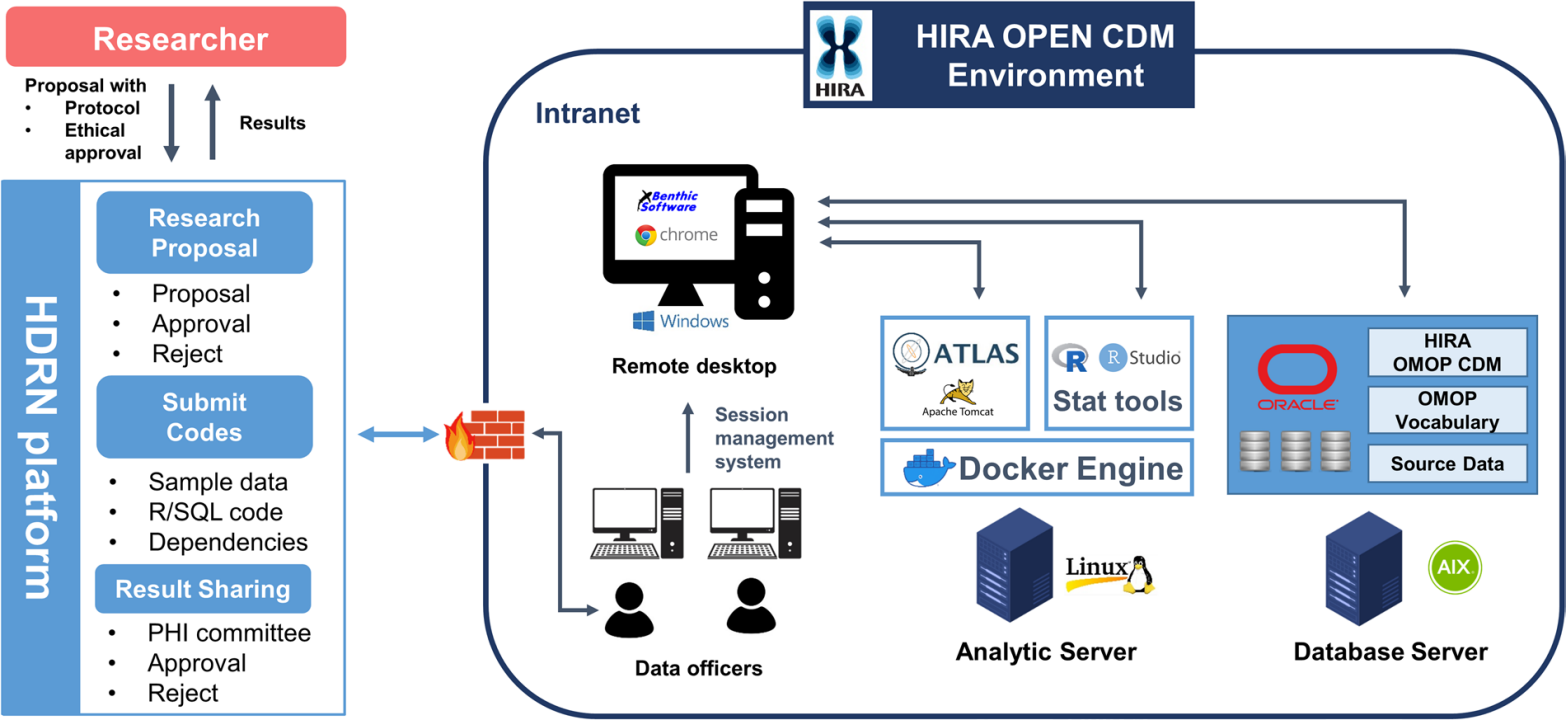
HIRA CDM analytic environment and data open process. Researchers can request the use of the HIRA CDM through the HDRN platform, which is an open public healthcare data platform. HDRN, Healthcare Distributed Research Network; PHI, personal health information; HIRA, Health Insurance Review and Assessment Service; CDM, common data model; OMOP, Observational Medical Outcome Partnership.

We built a Docker-based analytic environment for the use of open-source tools even in an intranet environment (offline for Internet) of the HIRA and to enable the installation of statistical tools and frequently updated packages (Fig. 2)^16^. For data security, the data officer of the HIRA is responsible for managing access sessions and logs from database and analytic servers.

By implementing the open policy of the HIRA CDM, researchers can apply for research requests through the healthcare distributed research network (HDRN) platform operated by the Korean government (https://hcdl.mohw.go.kr/). The specific application method is as follows: (1) The researcher must request a review of their research hypotheses and plan for ethical feasibility through an institutional or public review board. (2) The research must submit an approval letter from the review board and the research protocol to the HDRN platform. (3) The HIRA reviews the appropriateness of research/data provision and decides whether to provide it. (4) The researcher writes an analysis query, code, or package based on the open sample data and environment and sends it to the HIRA. (5) The HIRA reviews queries and expected results and derives results by running queries/codes/packages. (6) After the results are reviewed and the protected health information checked for infringement, the results are exported to the researcher.

We followed all FAIR principles, and the results of applying each principle to the HIRA CDM are shown in Table S10 in the Supplement. Metadata, disclosure policy, and sample data of HIRA CDM have been made available to the public online (https://opendata.hira.or.kr/op/opb/selectNotice.do?sno=13906&ntfcIteDivCd=&searchCnd=&searchWrd=cdm&pageIndex=1).

## Discussion

The HIRA CDM database is a useful national resource that encompasses abundant medical information of virtually all citizens and institutions in the Republic of Korea. An open research analysis system that complies with the FAIR principle was established to transparently utilize it for biomedical and healthcare research. While increasing researchers’ access to data resources, a distributed research system with privacy by design was established, such that national claims data across the country can be safely disclosed to external researchers without access to patient-level data. The established database and environment demonstrated the reproducibility and scalability of the research through comparative verification with source data and previously developed predictive models.

The Data Quality Dashboard^17^, the official quality assessment tool of OMOP CDM, was not performed because of limited hardware resources. This was because it was expected to take several months to be running to HIRA CDM, such a large size of data. In the comparison of T2DM incidence performed for quality assessment, there were differences between the original data and the CDM, however, which were attributed to changes in disease coverage during code conversion (source code to OMOP standardized vocabulary). This is an issue of mapping to different vocabulary systems and is not a data quality issue, however, researchers should be aware of such cases.

### Facilitating transparent and reproducible research

The retraction of COVID-19 research from high-profile journals underscores the necessity for open and reproducible science in healthcare^18^, which is particularly important for promoting confidence in science during the global health crisis. The current scientific landscape relies heavily on researchers’ reliability and trustworthiness. However, significantly high rates of data fabrication, falsification, and false-positive findings occur in healthcare research using big data for secondary use, further highlighting the necessity for more transparent research practices^19,20^. The usual policy of ‘sharing data upon request’ may not be optimal as it may limit the accessibility and usability of the data^21^. The common challenge against open science in healthcare is that patient-level data are inherently highly sensitive, making it difficult to share such data while preserving privacy. This challenge must be addressed by developing innovative approaches and technologies that can ensure safe and secure sharing of patient data while promoting open and reproducible science. Distributed research based on standardized data and vocabulary may guarantee reproducibility of research while preventing p-hacking.

### Scalable accessibility with privacy-by-design protection

Distributed research systems aimed toward data standardization guarantee scalable accessibility without privacy concerns because they enable privacy-by-design protection. Researchers cannot access patient-level data, and only anonymized data can be exported from the system to researchers. Despite being an internal environment with no Internet access, we utilized several open-source tools (most are Internet-dependent) to build our analytic environment. This unique approach uses the analytic codes or programs to perform the analysis instead of providing data to external researchers. Analytic queries, codes, and even a Docker-based analytic environment can be applied, enabling researchers to conduct reproducible analyses in the same local environment.

### Interoperability across countries

HIRA data can be used as a common data model such as OMOP-CDM in various approaches. Depending on the characteristics of the claims data, they include the life cycle information of the entire population; thus, expansion into various fields, such as the calculation of national statistics, research for clinical effectiveness, health care policy, and AI algorithms, is possible. The Republic of Korea is in the process of introducing OMOP-CDM to 57 medical institutions through past large national funding, suggesting that HIRA data can be utilized in association with the EHR-based databases of medical institutions using various methods. In addition, internationally, it is possible to cooperate with large-scale projects such as OHDSI, N3C^22^, EHDEN^23^, and DARWIN-EU^24^ based on the OMOP-CDM. Furthermore, as a national data infrastructure, it is possible to promote data harmonization with other data standards such as Fast Healthcare Interoperability Resources (e.g., http://omoponfhir.org/).

### FAIR research stewardship

As a custodian of nationwide healthcare data, the HIRA builds infrastructure for better research and data stewardship. Although data disclosure is important, the FAIR principle has rarely been applied to large-scale healthcare databases, owing to the sensitivity of personal data. In addition, the nature of the healthcare data provision process, in which researchers must rigorously vet data providers, often means that they do not provide sufficient information about the data. Providing metadata in accordance with FAIR can be part of a culture that improves access to information, and thus address information inequalities. For example, the structure of the database, original source of the data, time period of data, vocabulary, and application process for data access, etc.

## Methods

### Data source

HIRA claims data include complete information about medical services, such as patients’ visits to medical institutions, demographic information, medical service use, cost, disease conditions, and treatments including medications and procedures. The Republic of Korea introduced a mandatory national health insurance service to manage eligible citizens for health insurance from birth to death. In addition, a computerized system that enables the real-time linkage of medical records generated by medical institutions with the HIRA has been established. This study used the national claims data of the HIRA, which cover approximately 97% of the total population of the Republic of Korea (https://www.mohw.go.kr/eng/hs/hs0110.jsp?PAR_MENU_ID=1006&MENU_ID=100610). Furthermore, the HIRA data were linked to the national death registry of Statistics Korea; therefore, they were also included in this study. Data conversion and analyses were performed according to local laws and regulations and with approval from the respective scientific and ethics committees (Health Insurance Review and Assessment Institutional Review Board: 2022-014-001).

### Mapping to standardized vocabulary

Health insurance details (for diagnoses, medical fees, medication, and therapeutic materials) are reimbursed using the EDI code system in Korea; therefore, all details in the HIRA database are stored as EDI codes. We established a standard dictionary for the EDI code to construct the OMOP-CDM and integrated the EDI system into the OMOP standardized vocabulary through previous research^25^. Vocabulary mapping was conducted from terms for the reimbursement/non-reimbursement list of the EDI to the standard concepts for each domain according to OHDSI standardized vocabulary, e.g., diagnostic codes were mapped to SNOMED-CT, medication codes were mapped to RxNorm system (https://github.com/OHDSI/Vocabulary-v5.0/wiki/General-Structure-and-Use). Two or more healthcare experts independently conduct vocabulary mapping, and in case where their results differ, a third-party review makes the final decision. The final mapping list has been transparently disclosed online (Basic medical examination and diagnosis fee: https://opendata.hira.or.kr/op/opb/selectRfrm.do?rfrmTpCd=&searchCnd=&searchWrd=%EC%9A%A9%EC%96%B4&sno=13305&pageIndex=1 and Operation and Procedure fee: https://opendata.hira.or.kr/op/opb/selectNotice.do?searchCnd=&searchWrd=%EC%9A%A9%EC%96%B4&sno=13603&pageIndex=1).

Because standardized analysis using the OMOP-CDM is based on a standard vocabulary, if the ratio of unmapped records is high, information loss may occur because it cannot be used in the analysis. Code mapping and mapping record rates were checked to evaluate the possible information loss according to the vocabulary dictionary.

### Data conversion and quality assessment

**Fig. 3:**
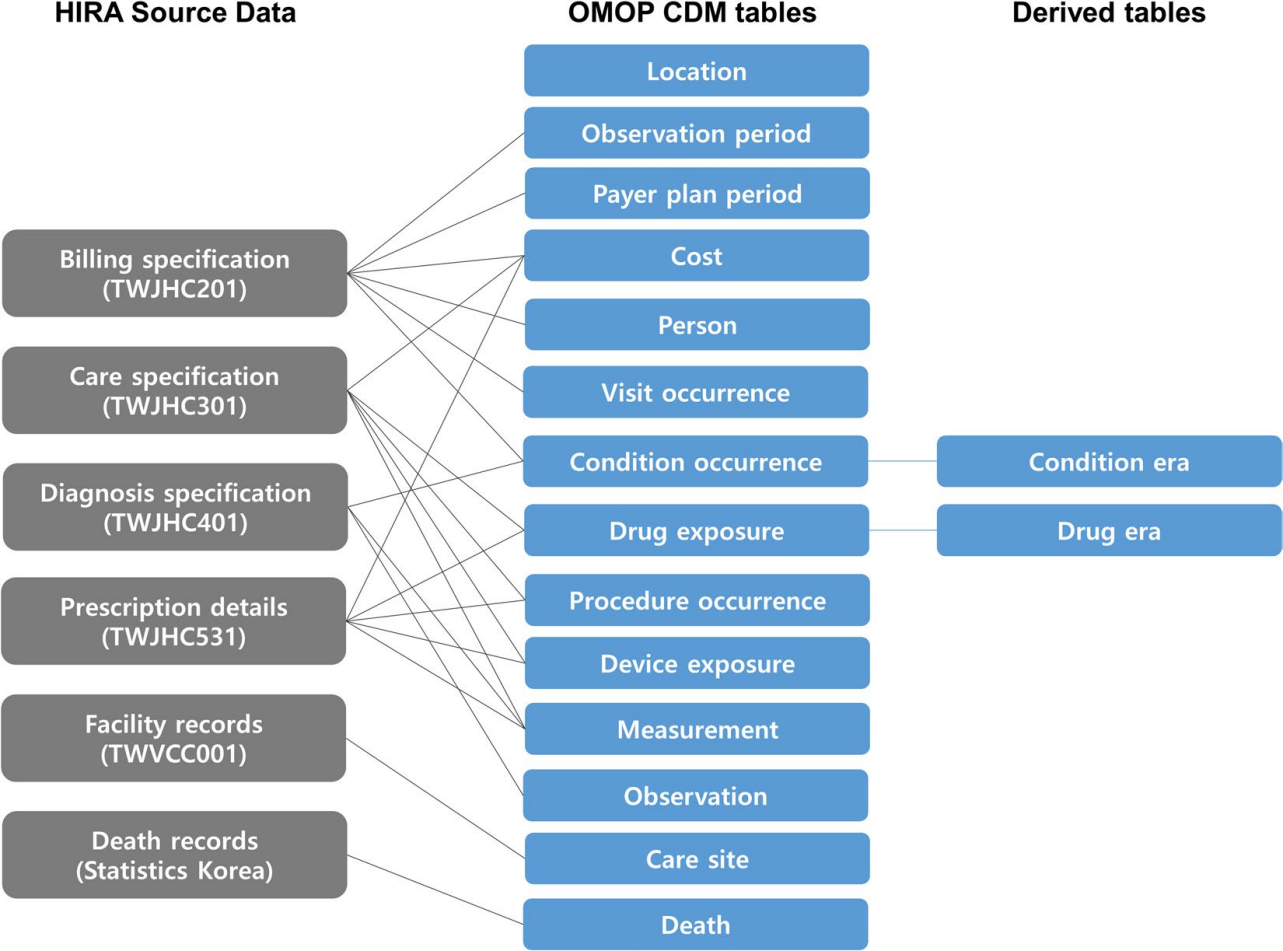
Data mapping to OMOP-CDM from HIRA source claims database. OMOP: Observational Medical Outcome Partnership; CDM: common data model; HIRA: Health Insurance Review and Assessment Service.

In this study, approximately 10 billion claim specifications for 56 million patients from 2010 to 2020 were converted into the OMOP-CDM. The data included information on healthcare institutions and death registry data, as well as general information, diagnosis, care, and prescription details of billing specifications. The source data of HIRA was converted by referring to the specification of OMOP-CDM version 5.3.1 (https://ohdsi.github.io/CommonDataModel/cdm53.html). Six types of source data were converted into 25 data tables of five table domains (clinical data, health system data, health economics data, standardized derived elements, metadata) and the data loaded with OMOP standardized vocabulary tables (Fig. 3). HIRA data were linked to the national death registry of Statistics Korea by national identification number. Under the current OMOP-CDM 5.3 convention, the death table was populated with the date of death and only one representative cause of death (underlying antecedent cause of death) for deceased patients. The pseudonymized patient identifiers and visit identifiers in the source data are maintained for consistency of the future conversion.

After the ETL process, we evaluated the quality of the HIRA CDM by assessing the concordance of descriptive statistics from the source and converted data. Statistical concordance between the source and HIRA CDM was evaluated. We compared the size of the data (by year and by type of medical service), number of medical institutions, and number of records with frequent codes within each domain. In addition, the number of patients with T2DM and its incidence in the middle of the year were calculated using the source and CDM databases. The digital phenotyping of T2DM was defined as those that had corresponding codes to E11-E14 of the International Classification of Disease (ICD-10) and A10 (‘*Drug used in diabetes’*) of Anatomical Therapeutic Chemical (ATC) Classification system^26^.

A previously published clinical prediction model was applied to corroborate the usability of the database and infrastructure established in this study. The COVER model was developed in the 2020 OHDSI COVID-19 study-a-thon, and the subset of HIRA database has already been used for the model validation study^15^. In the previous study, HIRA data included information of the patients with COVID-19 from 1 January to 4 April, 2020; however, in this study, we re-validated using data from two different databases: (1) the HIRA CDM database; 1 January, 2020, to 31 December, 2020, (2) 20% sampled database which newly updated information of the patients with COVID-19 until 30 April, 2022.

The target population was patients with COVID-19 infection and was defined as COVID-19 diagnosis or severe acute respiratory syndrome coronavirus 2 (SARS-COV-2) virus positive through the reverse transcription polymerase chain reaction (RT-PCR) test. The population was limited to adults (age ≥ 18) and without flu symptoms and pneumonia diagnosis within the previous 60 days. The outcomes to be predicted were as follows: (1) hospitalization for pneumonia within 30 days, (2) hospitalization for pneumonia requiring intensive care service or death after hospitalization for pneumonia from an index up to 30 days after the index, and (3) death within 30 days. The detailed model development process and evaluation method were performed in the same manner as described as in the previous publication.

### Infrastructure and data open policy

To utilize the HIRA CDM as a national data infrastructure, we established an open analytic environment and data access process for external researchers. To establish the analytic environment, our aim was to ensure that the analytic package developed by an external researcher using open-source tools (e.g., R) was sufficiently run, even in the closed intranet network of HIRA. We established a data acquisition process for external researchers, and the HIRA CDM data were disclosed according to the principle of distributed research using metadata and sample data. In all processes, we followed the FAIR principle, published the metadata online, and performed version control of the database.

### Supplementary information

Supplementary Information

## Data Availability

The authors declare that the data supporting the findings of this study are available within the paper and its supplementary information files. Data on vocabulary mapping were disclosed on the HIRA website (Basic medical examination and diagnosis fee: https://opendata.hira.or.kr/op/opb/selectRfrm.do?rfrmTpCd=&searchCnd=&searchWrd=%EC%9A%A9%EC%96%B4&sno=13305&pageIndex=1 and Operation and Procedure fee: https://opendata.hira.or.kr/op/opb/selectNotice.do?searchCnd=&searchWrd=%EC%9A%A9%EC%96%B4&sno=13603&pageIndex=1). According to Personal Information Protection Act in the Republic of Korea, HIRA does not permit us to share patient-level source data or data derivatives with individuals and institutions. The CDM data converted in this study is available as a distributed research network way upon an application through an online web portal (https://hcdl.mohw.go.kr). HIRA CDM is updated on an annual basis. Researchers can apply for research requests through the healthcare distributed research network (HDRN) platform operated by the Korean government. The specific application method is as follows: (1) The researcher must request a review of their research hypotheses and plan for ethical feasibility through an institutional or public review board. (2) The research must submit an approval letter from the review board and the research protocol to the HDRN platform. (3) The HIRA reviews the appropriateness of research/data provision and decides whether to provide it. (4) The researcher writes an analysis query, code, or package based on the open sample data and environment and sends it to the HIRA. (5) The HIRA reviews queries and expected results and derives results by running queries/codes/packages. (6) After the results are reviewed and the protected health information checked for infringement, the results are exported to the researcher. Detailed application process for data use is descripted in https://hcdl.mohw.go.kr/static/data/dataApplyStep.

## Code availability

We stored the CDM data using open-source codes of OHDSI for conforming to the database structure of OMOP CDM (https://github.com/OHDSI/CommonDataModel).
