# Supplementary material for: Scalable Infrastructure Supporting Reproducible Nationwide Healthcare Data Analysis toward FAIR Stewardship
Source: Sci Data. 2023 Oct 4;10:674. doi: 10.1038/s41597-023-02580-7 (PMC10550904; doi:10.1038/s41597-023-02580-7)
Supplement: Supplementary file 1 — Supplementary Information [file 41597_2023_2580_MOESM1_ESM.pdf]

Supplementary information

Supplement to: JW Kim, C Kim et al. Scalable Infrastructure Supporting Reproducible Nationwide Healthcare Data Analysis toward FAIR Stewardship.

Table of Contents

Table S1. Number of records by year, type of visit and type of medical institution in the source and CDM databases 2

Table S2. Number of records for the top 10 of conditions in the source and CDM databases 3

Table S3. Number of records for the top 10 of primary conditions in the source and CDM databases 4

Table S4. Number of records for the top 10 of procedure code in the source and CDM databases 5

Table S5. Number of records for the top 10 of drug code in the source and CDM databases 6

Table S6. Number of records for the top 10 of device code in the source and CDM databases 7

Table S7. Results of the number of patients with type 2 diabetes mellitus by year and gender 8

Table S8. Results of the number of patients with type 2 diabetes mellitus by year and age groups 9

Table S9. FAIR principles and HIRA open data strategy 13

Table S10. Performance results of COVER models using the HIRA databases having different data periods 12

REFERENCES 16

Table S1. Number of records by year, type of visit and type of medical institution in the source and CDM databases

Category	Source database (A)	CDM database (B)	Difference (A–B)
**Total, n**	10,098,730,241	10,098,730,241	0
**Year, n**			
2010	818,149,295	818,149,295	0
2011	836,105,372	836,105,372	0
2012	899,170,379	899,170,379	0
2013	909,869,673	909,869,673	0
2014	929,260,544	929,260,544	0
2015	924,941,727	924,941,727	0
2016	962,266,678	962,266,678	0
2017	963,926,363	963,926,363	0
2018	982,196,844	982,196,844	0
2019	1,001,317,069	1,001,317,069	0
2020	871,526,297	871,526,297	0
**Type of visit, n**			
Outpatient	9,915,565,629	9,915,565,629	0
Inpatient	183,164,605	183,164,605	0
Others	7	7	0
**Type of institution, n**			
Hospital	110,094	110,094	0
Long-term care facility	3,496	3,496	0
Dental hospital	33,433	33,433	0
Others	66,735	66,735	0

CDM: common data model

Table S2. Number of records for the top 10 of conditions in the source and CDM databases

Rank	ICD code	Name	Source data (A)	HIRA CDM (B)	Difference (A–B)
1	K29	Gastritis and duodenitis	2,044,449,281	2,044,449,281	0
2	J30	Vasomotor and allergic rhinitis	1,310,926,642	1,310,926,642	0
3	J20	Acute bronchitis	1,095,587,096	1,095,587,096	0
4	I10	Angina pectoris	839,267,192	839,267,192	0
5	M54	Dorsalgia	603,660,536	613,669,343	–10,008,807
6	E78	Disorder of lipoprotein metabolism and other lipidemias	595,275,724	595,275,724	0
7	K21	Gastro-esophageal reflux disease	480,293,477	480,293,477	0
8	K30	Functional dyspepsia	456,670,504	456,670,504	0
9	M79	Other and unspecified soft tissue disorders, not elsewhere classified	438,788,959	452,308,204	–13,519,245
10	E11	Type 2 diabetes mellitus	421,685,510	423,375,317	–1,689,807

CDM: common data model; ICD: international classification of disease 10th revision; HIRA: health insurance review and assessment service

Table S3. Number of records for the top 10 of primary conditions in the source and CDM databases

Rank	ICD code	Name	Source data (A)	HIRA CDM (B)	Difference (A–B)
1	J20	Acute bronchitis	556,586,441	556,586,441	0
2	I10	Essential (primary) hypertension	488,322,821	488,322,821	0
3	K05	Gingivitis and periodontal diseases	300,085,281	314,253,076	–14,167,795
4	M54	Dorsalgia	266,750,063	271,071,024	–4,320,961
5	M17	Osteoarthritis of knee	204,043,041	204,043,041	0
6	E11	Type 2 diabetes mellitus	198,701,427	199,371,732	–670,305
7	K04	Disease of pulp and periapical tissues	181,691,190	181,691,190	0
8	J30	Vasomotor and allergic rhinitis	166,255,214	166,255,214	0
9	J03	Acute tonsillitis	150,778,447	150,778,447	0
10	J06	Acute upper respiratory infections of multiple and unspecified sites	142,531,952	142,531,952	0

CDM: common data model; ICD: international classification of disease 10th revision; HIRA: health insurance review and assessment service

Table S4. Number of records for the top 10 of procedure code in the source and CDM databases

Rank	EDI code	Name	Source data (A)	HIRA CDM (B)	Difference (A–B)
1	AA254	Outpatient Care-Established Patient (Clinic)	4,253,071,311	4,253,071,311	0
2	AA154	Outpatient Care-New Patient	2,009,704,686	2,009,704,686	0
3	AL801	Medication Keeping Fee (Outpatient)	1,555,802,026	1,555,802,026	0
4	KK010	Subcutaneous or Intramuscular Injection	1,349,743,910	1,349,743,910	0
5	MM020	Deep Heat Therapy	741,156,685	741,156,685	0
6	MM015	Superficial Heat Therapy	730,597,720	730,597,720	0
7	D0002	Complete Blood Cell Count–Measuring equipment	594,885,915	277,936	594,607,979
8	AH200	Management of Chronic Disease	528,365,443	528,365,443	0
9	AA256	Outpatient Care-Established Patient (Hospital)	484,951,406	484,951,406	0
10	MM070	Transcutaneous Electrical Nerve Stimulation	476,389,412	476,389,412	0

CDM: common data model; EDI: electronic data interchange code; HIRA: health insurance review and assessment service

Table S5. Number of records for the top 10 of drug code in the source and CDM databases

Rank	EDI code	Name	Source data (A)	HIRA CDM (B)	Difference (A–B)
1	642102570	Chlorpheniramine maleate 2mg	660,651,573	660,651,573	0
2	643900710	Pseudoephedrine hydrochloride 60 mg	252,889,733	252,889,733	0
3	643501070	Streptokinase–streptodornase (streptokinase 10KI.U, streptodornase 2.5KI.U) 12.5KI.U	158,490,880	158,490,880	0
4	647500350	Dexibuprofen 0.4g	122,903,826	122,903,826	0
5	646900690	Acetaminophen(encapsulated) 0.65g	107,972,313	107,972,313	0
6	645701150	chlorpheniramine maleate 1.5mg,dihydrocodeine tartrate 5mg,DL-methylephedrine hydrochloride 17.5mg,guaifenesin 50mg	106,236,253	106,236,253	0
7	643501510	acetaminophen(encapsulated) 0.65g	102,575,178	102,575,178	0
8	642401650	piprinhydrinate 3mg	101,619,776	101,619,776	0
9	671802920	chlorpheniramine maleate 1.5mg,dihydrocodeine tartrate 5mg,DL-methylephedrine hydrochloride 17.5mg,guaifenesin 50mg	101,409,694	101,409,694	0
10	642502290	Artemisiae argyi folium 95% ethanol ext.(20→1) 60mg	95,196,995	95,196,995	0

CDM: common data model; EDI: electronic data interchange code; HIRA: health insurance review and assessment service

Table S6. Number of records for the top 10 of device code in the source and CDM databases

Rank	EDI code	Name	Source data (A)	HIRA CDM (B)	Difference (A–B)
1	L7250	Glass ionomer (Chemical polymerization)	47,442,404	47,442,404	0
2	K7202	Elastic bandage (10cm × 215cm)	40,228,319	40,228,319	0
3	K2062	Dental film (standard)	36,372,519	36,372,519	0
4	N0061	Ni-Ti File for Root-canal enlargement	28,658,980	28,658,980	0
5	K7201	Elastic bandage (15cm × 215cm)	27,997,908	27,997,908	0
6	K2056	Plain film (10 inch × 12 inch)	27,078,522	27,078,522	0
7	K2058	Plain film (8 inch × 10 inch)	25,764,488	25,764,488	0
8	K0001	Materials for Electrocardiography	24,558,659	24,558,659	0
9	N0041	Instruments for sub-endoscopic procedures	18,785,652	18,785,652	0
10	L7251	Glass ionomer (Chemical polymerization) Finished Product	18,683,892	18,683,892	0

CDM: common data model; EDI: electronic data interchange code; HIRA: health insurance review and assessment service

Table S7. Results of the number of patients with type 2 diabetes mellitus by year and gender

Year	Sex	Source database	HIRA CDM	Differences (A) – (B)	
		N (A)	N (B)	N	%*
2012	Male	186,070	185,680	390	(0.21)
	Female	140,662	140,462	200	(0.14)
2013	Male	173,266	173,030	236	(0.14)
	Female	131,720	131,564	156	(0.12)
2014	Male	166,560	166,495	65	(0.04)
	Female	125,277	125,255	22	(0.02)
2015	Male	175,044	175,363	-319	-(0.18)
	Female	129,171	129,421	-250	-(0.19)
2016	Male	194,724	194,490	234	(0.12)
	Female	143,209	143,071	138	(0.10)
2017	Male	202,668	202,558	110	(0.05)
	Female	147,843	147,769	74	(0.05)
2018	Male	209,192	209,105	87	(0.04)
	Female	147,378	147,315	63	(0.04)
2019	Male	218,685	218,650	35	(0.02)
	Female	155,489	155,465	24	(0.02)
2020	Male	222,966	222,964	2	(0.00)
	Female	161,538	161,526	12	(0.01)

*% $= \frac{\{(A)-(B)\}}{A} \times 100$; HIRA: health insurance review and assessment service; CDM: common data model

Table S8. Results of the number of patients with type 2 diabetes mellitus by year and age groups

Year	Age group	Source database	HIRA CDM	Differences (A) – (B)	
		N (A)	N (B)	N	%*
2012	0–19	1,662	1,678	-16	-(0.96)
	20–29	3,895	3,884	11	(0.28)
	30–39	20,072	20,033	39	(0.19)
	40–49	57,456	57,333	123	(0.21)
	50–59	93,463	93,296	167	(0.18)
	60–69	71,946	71,806	140	(0.19)
	70–79	57,412	57,304	108	(0.19)
	80–89	18,698	18,681	17	(0.09)
	90+	2,128	2,127	1	(0.05)
2013	0–19	1,690	1,702	-12	-(0.71)
	20–29	4,018	4,019	-1	-(0.02)
	30–39	19,639	19,619	20	(0.10)
	40–49	54,283	54,221	62	(0.11)
	50–59	88,073	87,958	115	(0.13)
	60–69	66,121	66,001	120	(0.18)
	70–79	51,823	51,745	78	(0.15)
	80–89	17,270	17,262	8	(0.05)
	90+	2,069	2,067	2	(0.10)
2014	0–19	1,842	1,865	-23	-(1.25)
	20–29	4,268	4,269	-1	-(0.02)
	30–39	19,437	19,440	-3	-(0.02)
	40–49	52,069	52,020	49	(0.09)
	50–59	83,815	83,787	28	(0.03)
	60–69	63,329	63,296	33	(0.05)
	70–79	48,163	48,149	14	(0.03)
	80–89	16,908	16,913	-5	-(0.03)
	90+	2,006	2,011	-5	-(0.25)

2015	0–19	1,904	1,917	-13	-(0.68)
	20–29	4,485	4,492	-7	-(0.16)
	30–39	20,118	20,177	-59	-(0.29)
	40–49	53,409	53,521	-112	-(0.21)
	50–59	87,754	87,923	-169	-(0.19)
	60–69	69,107	69,241	-134	-(0.19)
	70–79	47,682	47,740	-58	-(0.12)
	80–89	17,683	17,703	-20	-(0.11)
	90+	2,073	2,070	3	(0.14)
2016	0–19	2,498	2,510	-12	-(0.48)
	20–29	5,294	5,279	15	(0.28)
	30–39	22,453	22,409	44	(0.20)
	40–49	58,617	58,551	66	(0.11)
	50–59	97,444	97,310	134	(0.14)
	60–69	79,175	79,068	107	(0.14)
	70–79	51,210	51,186	24	(0.05)
	80–89	19,106	19,110	-4	-(0.02)
	90+	2,136	2,138	-2	-(0.09)
2017	0–19	2,436	2,447	-11	-(0.45)
	20–29	5,550	5,545	5	(0.09)
	30–39	23,348	23,340	8	(0.03)
	40–49	59,386	59,364	22	(0.04)
	50–59	99,207	99,147	60	(0.06)
	60–69	83,704	83,652	52	(0.06)
	70–79	53,837	53,802	35	(0.07)
	80–89	20,657	20,642	15	(0.07)
	90+	2,386	2,388	-2	-(0.08)
2018	0–19	2,660	2,656	4	(0.15)
	20–29	6,283	6,274	9	(0.14)
	30–39	24,494	24,491	3	(0.01)
	40–49	58,835	58,805	30	(0.05)

	50–59	100,558	100,522	36	(0.04)
	60–69	86,609	86,571	38	(0.04)
	70–79	53,484	53,465	19	(0.04)
	80–89	21,144	21,134	10	(0.05)
	90+	2,503	2,502	1	(0.04)
2019	0–19	2,562	2,581	-19	-(0.74)
	20–29	6,998	6,988	10	(0.14)
	30–39	25,665	25,660	5	(0.02)
	40–49	59,925	59,914	11	(0.02)
	50–59	102,825	102,806	19	(0.02)
	60–69	93,243	93,229	14	(0.02)
	70–79	56,567	56,559	8	(0.01)
	80–89	23,391	23,380	11	(0.05)
	90+	2,998	2,998	0	(0.00)
2020	0–19	2,584	2,596	-12	-(0.46)
	20–29	7,685	7,692	-7	-(0.09)
	30–39	26,035	26,030	5	(0.02)
	40–49	60,752	60,752	0	(0.00)
	50–59	103,779	103,769	10	(0.01)
	60–69	100,147	100,142	5	(0.00)
	70–79	57,121	57,113	8	(0.01)
	80–89	23,591	23,589	2	(0.01)
	90+	2,810	2,807	3	(0.11)

*% = $\frac{\{(A)-(B)\}}{A} \times 100$; HIRA: health insurance review and assessment service; CDM: common data model

Table S9. Performance results of COVER models using the HIRA databases having different data periods

Data period	Population (n)	Outcome (n, [%])	AUC	AUPRC
–2020 Apr*	Covid-19 infection (1 985)	Hospitalization for pneumonia (89 [4.48%])	0.806	0.134
		ICU or death due to pneumonia (22 [1.11%])	0.910	0.053
		All cause death (43 [2.17%])	0.898	0.150
–2020 Dec	Covid-19 infection (32 633)	Hospitalization for pneumonia (1 250 [3.83%])	0.816	0.149
		ICU or death due to pneumonia (188 [0.58%])	0.891	0.036
		All cause death (205 [0.63%])	0.892	0.052
–2022 Apr	Covid-19 infection (1 530 350)	Hospitalization for pneumonia (4 806 [0.31%])	0.748	0.013
		ICU or death due to pneumonia (487 [0.03%])	0.879	0.003
		All cause death (1 190 [0.08%])	0.891	0.012

*Results from data (–2020 Apr) are cited from previously published paper (Williams, R.D et al).[1]

COVER: Covid-19 Estimated Risk; HIRA: health insurance review and assessment service; Covid-19: coronavirus disease 2019; ICU: intensive care unit; AUC: area under the receiver operating characteristics curve; AUPRC: area under the precision-recall curve.

Table S10. FAIR principles and HIRA open data strategy

<table>
<thead>
<tr><th>FAIR principles</th><th>Strategy of HIRA</th></tr>
</thead>
<tbody>
<tr><td>Findable</td><td></td></tr>
<tr><td>F1. (Meta)data are assigned a globally unique and persistent identifier</td><td>The source data of the HIRA CDM is the claims details collected in accordance with the National Health Insurance Act. The information collected includes personal identification numbers (resident registration numbers) managed by the government and medical institution numbers. Since related identifiers are managed by the country, they can be used uniquely and persistently. OMOP–CDM uses OMOP standardized vocabulary, so identifiers for all records other than individual patient identifiers are globally unique, persistent, and disclosed (https://athena.ohdsi.org/).</td></tr>
<tr><td>F2. Data are described with rich metadata(defined by R1 below)</td><td>The structure of HIRA CDM is same with the OMOP–CDM version 5.3. The meta data of OMOP–CDM version 5.3 is in online repository (https://ohdsi.github.io/CommonDataModel/cdm53.html)</td></tr>
<tr><td>F3. Metadata clearly and explicitly include the identifier of the data they describe</td><td>The database is built according to the structure of OMOP-CDM version 5.3, and the data structure and column names of the HIRA CDM database can be checked using the metadata of OMOP-CDM. In addition, metadata, table schema, and sample data are disclosed so that external researchers can fully understand the structure of the data.</td></tr>
<tr><td>F4. (Meta)data are registered or indexed in a searchable resource</td><td>If you search for “healthcare distributed research network” on Google, you can find the HDRN webpage, and if you search for “hdrn hira cdm”, you will find a link to a page where you can download sample data of HIRA CDM provided by HDRN.
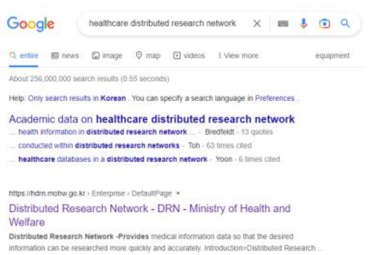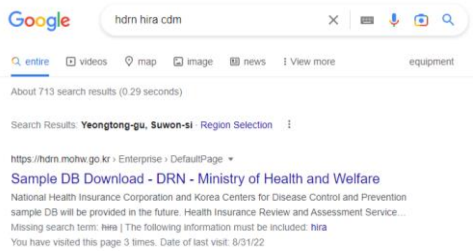</td></tr>
<tr><td>Accessible</td><td></td></tr>
<tr><td>A1. (Meta)data are retrievable by their identifier using a standardised communications protocol</td><td>Information on the HIRA database can be found on the HDRN website (https://hdrn.mohw.go.kr), and all websites can be accessed through HTTPS.</td></tr>
</tbody>
</table>

A1.1 The protocol is open, free, and universally implementable	Information on the HIRA database can be found on the HDRN website (https://hdrn.mohw.go.kr), which can be accessed for free.
A1.2 The protocol allows for an authentication and authorisation procedure, where necessary	No special authentication procedure is required for HDRN website access, if necessary, authorization is performed through the login process.
A2. Metadata are accessible, even when the data are no longer available	Meta information and sample data are maintained through version management even for databases that are not in use.
**Interoperable**	
I1. (Meta)data use a formal, accessible, shared, and broadly applicable language for knowledge representation	To ensure automatic findability and interoperability of datasets, HIRA CDM use controlled vocabularies (well-known, broadly adopted medical ontologies) and fully specified data model (OMOP–CDM).
I2. (Meta)data use vocabularies that follow FAIR principles	The vocabulary used in HIRA CDM is an OMOP standardized vocabulary which house existing vocabularies used in the public domain, and details such as definition and id rules are disclosed in the wiki page (https://github.com/OHDSI/Vocabulary-v5.0/wiki), and the vocabulary can be searched and downloaded in the portal (https://athena.ohdsi.org/).
I3. (Meta)data include qualified references to other (meta)data	Information on OMOP–CDM was described on the HDRN homepage, and a link was attached so that qualified information on the OMOP–CDM could be obtained.<br>Common Data Model (CDM)<br>• The Common Data Model (CDM) refers to a data model that standardizes the different data structures and meanings of each partner organization into a standardized structure to have the same structure and meaning.<br>• The Common Data Model (CDM) standardizes data models, terminology, analysis methods, methodologies, etc., and establishes the basis for RWE (Real World Evidence) , such as repeatable, reproducible, reproducible, generalizable, robust, and correctable, for existing analysis complexity can be eliminated.<br>• There are various types of Common Data Model (CDM) , such as Sentinel CDM, PCORnet CDM, and OMOP (Observational Medical Outcome Partners) CDM .<br>- OMOP-CDM is used in health care research on various topics such as drug/device side effect study, comparative effect study, economic analysis, medical quality evaluation, clinical study, etc. For more information on OMOP CDM, please refer to the OHDIS website.<br>https://www.ohdsi.org/data-standardization/the-common-data-model/<br>https://hdrn.mohw.go.kr/EXDRN/Portal/Enterprise/DefaultPage.bzr?tabID=1020&ftab=1003

**Reusable**	
R1. (Meta)data are richly described with a plurality of accurate and relevant attributes	The HIRA discloses sample data and documents of HIRA CDM including sufficient meta information (definition of patients, data period, gender/age standardization (yes/no), including sensitive diseases (yes/no), etc.). The sample data of HIRA CDM is also provided in the HDRN platform (URL: https://hdrn.mohw.go.kr).

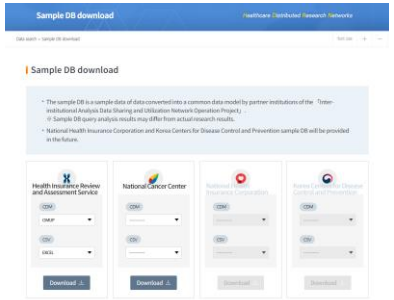

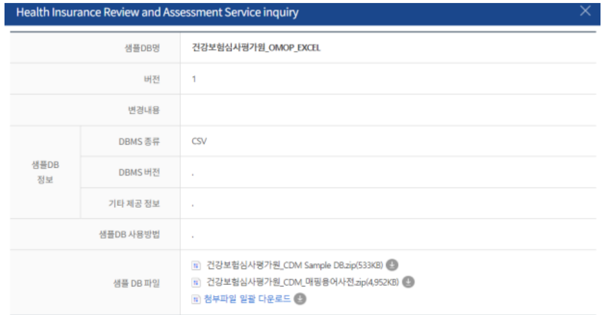

R1.1. (Meta)data are released with a clear and accessible data usage license	OMOP–CDM follows Apache 2.0 license, and Athena including standardized vocabularies follows the Unlicense. HIRA CDM is not open itself to the outside, and research is conducted by sharing analysis packages with Apache 2.0 licenses from external researchers.
R1.2. (Meta)data are associated with detailed provenance	Research results analyzed through the HIRA CDM should include information about data sources in Methods and information about data provision by HIRA in Acknowledgment within the research publication.
R1.3. (Meta)data meet domain-relevant community standards	The data structure of HIRA CDM is fully conformed with OMOP–CDM version 5.3.1 and the detailed specification can be found in the GitHub wiki page and repository.<br>Wiki: https://ohdsi.github.io/CommonDataModel/cdm53.html<br>Data definition language (DDL) GitHub repository :<br>https://github.com/OHDSI/CommonDataModel/blob/v5.3.1/Oracle/OMOP%20CDM%20oracle%20ddl.txt

FAIR: Findability, Accessibility, Interoperability, and Reuse of digital assets; HIRA: health insurance review and assessment service.

REFERENCES

1. Williams, R.D., Markus, A.F., Yang, C. *et al.* Seek COVER: using a disease proxy to rapidly develop and validate a personalized risk calculator for COVID-19 outcomes in an international network. *BMC Med Res Methodol* **22**, 35 (2022). https://doi.org/10.1186/s12874-022-01505-z
